# The Role of Fungal Microbiome Components on the Adaptation to Salinity of *Festuca rubra* subsp. *pruinosa*

**DOI:** 10.3389/fpls.2021.695717

**Published:** 2021-07-09

**Authors:** Eric C. Pereira, Beatriz R. Vazquez de Aldana, Juan B. Arellano, Iñigo Zabalgogeazcoa

**Affiliations:** Plant-Microorganism Interaction Research Group, Institute of Natural Resources and Agrobiology of Salamanca, Consejo Superior de Investigaciones Científicas (IRNASA-CSIC), Salamanca, Spain

**Keywords:** endophytes, *Epichloë*, fungi, *Fusarium oxysporum*, halophyte, *Periconia macrospinosa*, salinity, symbiosis

## Abstract

*Festuca rubra* subsp. *pruinosa* is a perennial grass that inhabits sea cliffs, a habitat where salinity and low nutrient availability occur. These plants have a rich fungal microbiome, and particularly common are their associations with *Epichloë festucae* in aboveground tissues and with *Fusarium oxysporum* and *Periconia macrospinosa* in roots. In this study, we hypothesized that these fungi could affect the performance of *F. rubra* plants under salinity, being important complements for plant habitat adaptation. Two lines of *F. rubra*, each one consisting of *Epichloë*-infected and *Epichloë*-free clones, were inoculated with the root endophytes (*F. oxysporum* and *P. macrospinosa*) and subjected to a salinity treatment. Under salinity, plants symbiotic with *Epichloë* had lower Na^+^ content than non-symbiotic plants, but this effect was not translated into plant growth. *P. macrospinosa* promoted leaf and root growth in the presence and absence of salinity, and *F. oxysporum* promoted leaf and root growth in the presence and absence of salinity, plus a decrease in leaf Na^+^ content under salinity. The growth responses could be due to functions related to improved nutrient acquisition, while the reduction of Na^+^ content might be associated with salinity tolerance and plant survival in the long term. Each of these three components of the *F. rubra* core mycobiome contributed with different functions, which are beneficial and complementary for plant adaptation to its habitat in sea cliffs. Although our results do not support an obvious role of *Epichloë* itself in FRP salt tolerance, there is evidence that *Epichloë* can interact with root endophytes, affecting host plant performance.

## Introduction

**Figure 1:**
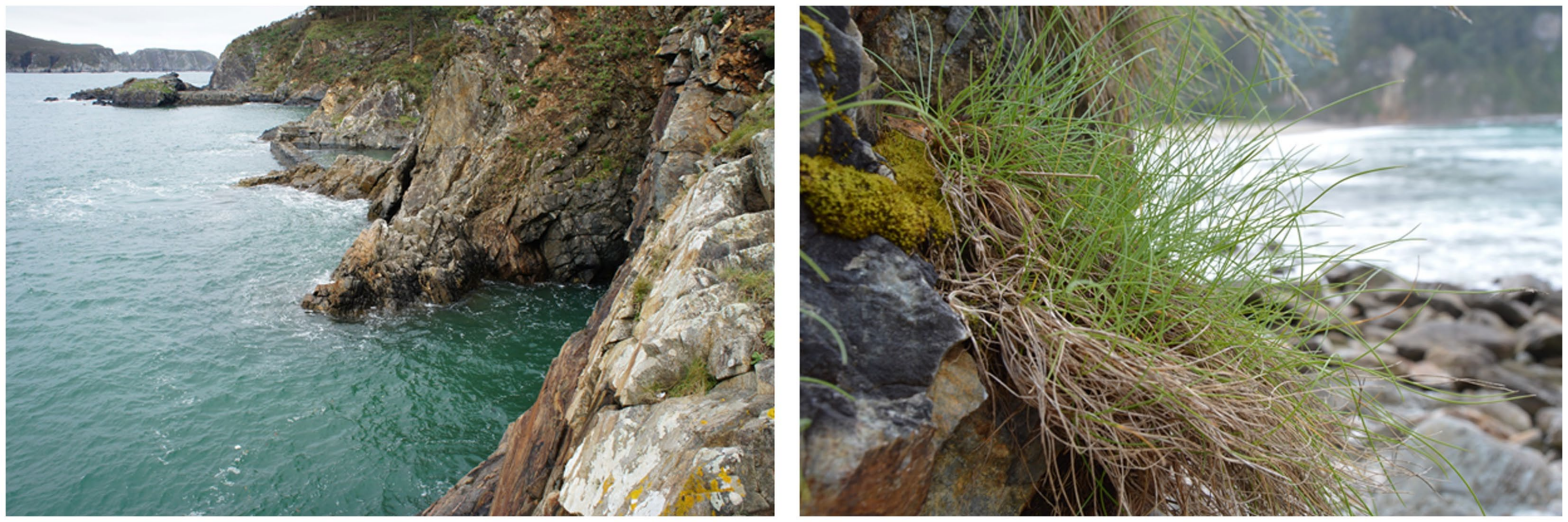
*Festuca rubra* subsp. *pruinosa* (FRP) inhabits rocky sea cliffs of the Atlantic coasts of Europe (left). Plants often grow in rock fissures, where soil is absent, and are very exposed to wind and saline sea spray (right).

*Festuca rubra* is a perennial grass that occurs in very diverse ecological niches (Markgraf-Dannenberg, 1980; Inda et al., 2008). In addition to its value as a forage, commercial cultivars of this species are used for ornamental and sports lawns (Braun et al., 2020). Within *F. rubra*, several subspecies have been defined and three of them, *litoralis*, *pruinosa*, and *arenaria*, occur in maritime habitats such as salt marshes, sea cliffs, or coastal sands (Markgraf-Dannenberg, 1980). As a result of habitat adaptation, maritime subspecies are more tolerant to salinity than inland subspecies (Hannon and Bradshaw, 1968; Rozema et al., 1978). *Festuca rubra* subsp. *pruinosa* (FRP) inhabits sea cliffs of the Atlantic coasts of Europe (Figure 1; Markgraf-Dannenberg, 1980). This grass often grows as a chasmophyte in rock crevices with low nutrient availability and high exposure to seawater spray and desiccating winds. Some structural traits seemingly associated with salt tolerance in FRP are a dense layer of epicuticular wax which covers its leaves, stomata enclosed on the adaxial side of c-sectioned leaves, together with a thickened root endodermis in comparison with inland fescues (Baumeister and Merten, 1981; Ortuñez and de la Fuente, 2010; Martinez-Segarra et al., 2017).

In addition to the plant traits that may favor tolerance to salinity and water loss, or improve nutrient absorption in a suboptimal environment, the plant microbiome can also provide auxiliary functions that facilitate the habitat adaptation of holobionts (i.e., the host plant and its microbiota; Rodriguez et al., 2008; Vandenkoornhuyse et al., 2015; Trivedi et al., 2020). In a previous study, 135 different fungal taxa were identified as culturable components of the fungal mycobiome of FRP roots (Pereira et al., 2019). *Fusarium oxysporum* was the most abundant taxon, being found at all populations sampled and in 57% of the plants analyzed*. Periconia macrospinosa*, a dark septate endophyte (DSE), was also an abundant component of the root microbiome, and it was found in 16% of the plants. In addition, aerial tissues of 66% of the FRP plants were colonized by the fungal endophyte *Epichloë festucae*. As possible components of the core microbiome of FRP, we here hypothesize that these fungi (*F. oxysporum, P. macrospinosa*, and *E. festucae*) could provide functions related to plant adaptation to the sea cliff environment.

*Epichloë festucae* asymptomatically colonizes the intercellular space of stem and leaf tissues of *F. rubra* and other grasses, and is vertically transmitted to seeds (Clay and Schardl, 2002; Leuchtmann et al., 2014). This fungal endophyte might produce antiherbivore secondary metabolites, such as the bioactive alkaloid ergovaline in symbiotic FRP plants (Vázquez de Aldana et al., 2007). Although *Epichloë* endophytes are not present in plant roots, their presence in aboveground plant tissues can affect several belowground processes (Omacini et al., 2012; Rojas et al., 2016). *Epichloë* is one of the best known taxa of endophytic fungi in aboveground tissues, but for most components of root mycobiomes, their functions as plant symbionts, as well as their life cycles, are unknown (Pozo et al., 2021).

Soil salinity inhibits plant growth and development by reducing water uptake, and also induces cytotoxicity due to excess of Na^+^ ions, oxidative stress due to the generation of reactive oxygen species, and nutritional imbalance (Munns and Tester, 2008; Zhao et al., 2020). Plants, particularly halophytes, have mechanisms involved in adaptation to salinity, like Na^+^ exclusion, Na^+^ intracellular accumulation and osmoregulation, or alteration of the level of secondary metabolites, including phenolic compounds and their antioxidant capacity (Munns and Tester, 2008; Waśkiewicz et al., 2013; van Zelm et al., 2020; Zhao et al., 2020). In addition, there is compelling evidence of fungal microbiome components contributing to plant adaptation to salinity. For example, some strains of *Epichloë*, *Diaporthe*, *Piriformospora*, *Penicillium*, *Fusarium*, and DSE have been reported to improve plant growth under salinity (Waller et al., 2005; Rodriguez et al., 2008; Molina-Montenegro et al., 2018; Pereira et al., 2019; Gonzalez Mateu et al., 2020; Wang et al., 2020).

The main purpose of this study was to explore the effect of the foliar endophyte *E. festucae* and the root endophytes *F. oxysporum* and *P. macrospinosa* on the performance of FRP plants subjected to salinity. We present data supporting that these microbiome components could be involved in the host plant adaptation to its maritime habitat.

## Materials and Methods

### Plant and Fungal Material

Two near-isogenic lines of FRP (TH12 and EB15) were used to test the effects of *E. festucae* and two root endophytes on plant performance. Each line consisted of a unique plant genotype infected (E^+^) or not infected (E^–^) by a unique *E. festucae* genotype. Each line was generated from a single E^+^ FRP plant originally collected in the coast of Galicia (Figure 1; Pereira et al., 2019). This plant was split into several clones that were transplanted to 200 ml pots containing a 1:1 (v:v) mixture of peat and perlite. To obtain E^–^ plants, one half of the E^+^ clones were treated with the systemic fungicide propiconazole to eliminate the fungus (Zabalgogeazcoa et al., 2006a). Six doses of 400 μg of propiconazole were applied to each plant: The first, fourth, fifth, and sixth doses were applied to the soil, and the second and third were foliar applications. Fungicide treatments were spaced by 5 days for the first three treatments and by 10 days for the last three soil applications. Four weeks after the last dose, newly formed ramets from each clone were obtained, and their E^+^ or E^−^ status was verified by direct isolation of *E. festucae* from surface-disinfected leaf sheaths (Florea et al., 2015). Two different near-isogenic lines of FRP were used because different *Epichloë*/grass genotypes might differ in terms of stress responses, nutrient accumulation, or alkaloid content (Cheplick et al., 2000; Zabalgogeazcoa et al., 2006a; Vázquez de Aldana et al., 2020b). Only one root endophyte was inoculated into each *Festuca* line because of limited plant clone availability.

The fungal strains, *F. oxysporum* T48 and *P. macrospinosa* T131, were originally isolated as endophytes from surface-disinfected roots of asymptomatic FRP plants collected in natural populations in the northern coast of Galicia, Spain (Pereira et al., 2019). These strains were selected because they belonged to two of the most abundant taxa from the culturable fungal microbiome of roots from apparently healthy FRP plants (Pereira et al., 2019). Thus, we assumed that these microbiome components were non-pathogenic and could have a role in holobiont adaptation. In addition, *F. oxysporum* strains from FRP are non-pathogenic on tomato plants, as shown by Constatin et al. (2020).

### Effect of *Epichloë* and Root Endophytes on Plant Growth Under Salinity

To determine the effect of *F. oxysporum* T48 and *P. macrospinosa* T131 (onward *F. oxysporum* and *P. macrospinosa*) on the growth of E^+^ and E^−^ FRP plants, a greenhouse experiment was designed. Plants of the line EB15 were inoculated with *F. oxysporum*, and those of the line TH12 with *P. macrospinosa*. Each clone to be inoculated with a root endophyte was transplanted to a 200 ml pot containing a substrate composed of seven parts (v:v) of a mixture of peat and perlite (1:1 v/v) previously treated at 80°C for 24 h, and one part of fungal inoculum prepared in a beet pulp medium for 4 weeks (Vázquez de Aldana et al., 2020a). Non-inoculated clones were transplanted to pots containing only the peat and perlite substrate. A three-factor experiment was carried out for each line, with *Epichloë* infection (E^+^ or E^–^), root endophyte inoculation with *F. oxysporum* or *P. macrospinosa* (uninoculated or inoculated), and salinity (watering with 0 or 600 mM NaCl), with five plant replicates per treatment. Plants subjected to the salinity treatment were watered with 200 mM NaCl on the first day to avoid salt shock and with 600 mM NaCl afterward for 5 weeks. After this time, the whole plants were harvested, roots were carefully washed with tap water, and some pieces from each plant were kept for observation by optical microscopy. The plants were lyophilized, and their dry biomass was recorded. The aboveground parts, which consisted of leaves and leaf sheaths, were ground and used for chemical analysis.

To check for the presence of *F. oxysporum* or *P. macrospinosa* in roots of all inoculated plants, fresh root fragments were cleared in 5% KOH at 90°C for 15 min, neutralized with approximately three volumes of 1% HCl at 20°C overnight, stained with trypan blue (Berthelot et al., 2016), and visualized by light microscopy.

### Sodium, Potassium, and Proline Content

To estimate the concentration of mineral elements, leaf samples (five replicates of each treatment) were calcined at 450°C for 8 h and ashes were dissolved in HCl:HNO_3_:H_2_O (1:1:8). Na and K contents were analyzed by inductively coupled plasma atomic emission spectroscopy (ICP-OES) in a Varian 720-ES (Agilent, United States) spectrometer.

Leaf proline content was quantified in three plant replicates of each treatment using the spectrophotometric method described by Shabnam et al. (2016), adapted to 96-well plates in our laboratory. Approximately 15 mg of plant material was homogenized in 500 μl of 3% aqueous 5-sulfosalicylic acid and kept for 10 min in ice. The mixture was centrifuged at 10°C and 16,000 *g* for 10 min, and the supernatant was mixed with 250 μl of glacial acetic and 500 μl of ninhydrin reagent. Then, the mixture was heated at 99°C for 40 min and immediately cooled in ice. The mixture was centrifuged, and an aliquot of 200 μl transferred to a 96-well plate where the absorbance was measured at 513 nm in a FLUOstar Omega plate reader (BMG Labtech, Germany). L(−) proline (Acrós Organics) was used as standard for quantification.

### Ferric Reducing Antioxidant Potential Assay

The total antioxidant capacity was determined in leaves of five replicates of each treatment using the ferric ion reducing antioxidant power (FRAP) method (Benzie and Strain, 1996). This method is based on the reduction of the colorless [Fe(III)−,4,6-tri(2-pyridyl)-*s*-triazine)_2_]^3+^ complex, abbreviated as Fe(III)-TPTZ, to the blue-colored Fe(II)-TPTZ complex, formed by the action of electron donating antioxidants at low pH. The FRAP reagent was prepared by mixing 300 mM acetate buffer pH = 3.6, a solution of 10 mM 2,4,6-tripyridyl-s-triazine (TPTZ) in 40 mM HCl, and 20.35 mM FeCl_3_ at a volume ratio of 10:1:1. 5 mg of each plant sample was extracted in 700 μl of 50% aqueous acetone for 30 min in an ultrasound bath at 8°C. The mixture was centrifuged and transferred to a 96-well plate where 8 μl of sample, 8 μl of phosphate buffer saline (PBS), and 200 μl of FRAP reagent were added to each well. The absorbance was measured at 593 nm after 30 min in a FLUOstar Omega (BMG Labtech, Germany). A standard curve was prepared using different concentrations of 6-hydroxy-2,5,7,8-tetramethylchroman-2-carboxylic acid (Trolox). The TPTZ solution was freshly prepared before use. The results were expressed as μmol Trolox equivalent per gram of dry weight.

### Phenolic Compounds Content

The content of total phenolic compounds in leaf samples was determined spectrophotometrically according to the Folin-Ciocalteu method (Ainsworth and Gillespie, 2007). For the analyses, five replicates of each treatment were used. A 100 μl aliquot of acetone extract of each sample, prepared as previously described for the FRAP assay, was mixed with 500 μl of Folin-Ciocalteu reagent (Scharlab Chemie SA). After 5 min, a volume of 400 μl of a 700 mM Na_2_CO_3_ solution was added. The mixture was incubated for 60 min, and the absorbance at 765 nm was measured in a 96-well plate in a FLUOstar Omega (BMG Labtech, Germany). Gallic acid was used as a reference standard, and the results were expressed as μmol gallic acid equivalent per gram of dry weight.

### Ergovaline Content

The *Epichloë* alkaloid ergovaline was analyzed in leaf samples of E^+^ plants (three replicates of each treatment) by HPLC following a modification of the methods described by Hill et al. (1993) and Yue et al. (2000). Extraction was conducted in 0.5 g of plant material, adding 10 ml of chloroform, 0.5 ml of a methanolic solution 5 mM NaOH, and an internal standard of ergotamine (Sigma-Aldrich). The mixture was placed on an orbital shaker at 100 rpm for 120 min, paper-filtered (Filter Lab 1240), washed with 3.0 ml of chloroform, and then passed through an Ergosil – HL silica gel (500 mg, Analtech) column preconditioned with 5.0 ml of chloroform. To eliminate pigments, a solution of 5.0 ml chloroform:acetone (75:25 v/v) was passed through the column, and the sample was eluted with 3.0 ml of methanol and dried under a nitrogen stream. The residue was dissolved in 1.0 ml of methanol and filtered through a 0.45 μm nylon disk. Ergovaline quantification was performed in a Waters 2695 HPLC system, with a C18 column (150 × 4.6 mm; 2.7 μm; Agilent Poroshell) and a fluorescence detector (Waters 2475). The excitation and emission wavelengths were 250 nm and 420 nm, respectively. The mobile phase was acetonitrile: 0.01 M ammonium acetate (3:7) with a gradient flow of 0.6 ml/min. The gradient was adjusted through time programming as follows: step 1, 30% acetonitrile and 70% ammonium acetate for 18 min; step 2, 45%:55% for 2 min; step 3, 85%:15% for 2 min; step 4, 100% acetonitrile for 5 min; step 5, 100% acetonitrile for 3 min; and step 6, 30%:70% for 2 min. The ergovaline standard was purchased from Forrest Smith (Auburn University, United States).

### *In vitro* Dual-Culture Interaction

A dual-culture assay was made to analyze *in vitro* interactions between *E. festucae* and both root endophytes. The *E. festucae* strain from E^+^ plants of the line TH12 was co-cultured with *P. macrospinosa*, and the *E. festucae* strain from EB15 plants with *F. oxysporum*. Mycelial disks (6 mm diameter) from PDA cultures of each fungus were placed 4 cm apart on the surface of 9 cm Petri dishes containing PDA medium. Controls consisted of agar plates containing two disks of the same strain. Five replicates of each combination were incubated at room temperature in the dark. The evolution of the radii of the interacting strains was measured for several days. Interactions between strains were assessed based on the inhibition or non-inhibition of their mycelial growth.

### Statistical Analyses

The effects of *E. festucae*, root endophyte inoculation, and salinity on biomass, Na^+^ and K^+^ content, proline, total phenolic compounds, and antioxidant activity were analyzed by means of a three-way ANOVA. For ergovaline concentration in E^+^ plants, the effects of root endophyte inoculation and salt treatment were analyzed with a two-way ANOVA. The data sets were evaluated for the statistical assumptions of the ANOVA with the Shapiro-Wilk normality test and Brown-Forsythe equal variance test. Differences between means of estimated effects of significant factors and their interaction were evaluated using Tukey’s test (*p* < 0.05). All statistical analyses were performed with SigmaPlot v.14.

## Results

### Biomass Production

**Figure 2:**
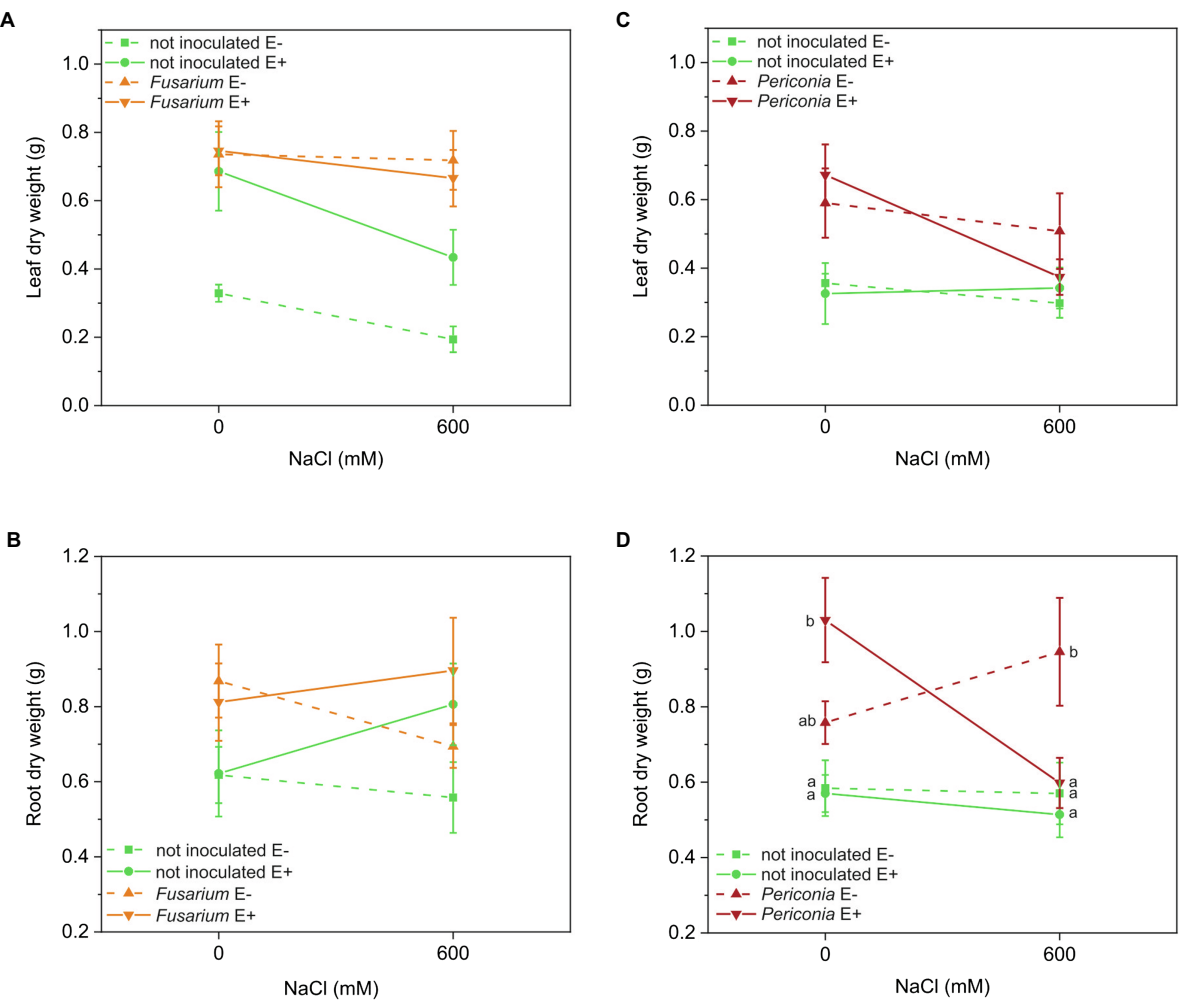
Leaf and root biomass produced at two different salinity treatments (0 and 600 mM NaCl) in two FRP lines, EB15 (A,B) and TH12 (C,D), each one composed by clones symbiotic with Epichloë festucae (E^+^) or Epichloë free (E^–^), and inoculated with Fusarium oxysporum T48 (orange), Periconia macrospinosa T131 (dark red), or uninoculated (green). Where a significant [salinity × Epichloë × root endophyte] interaction occurred, different means are indicated by different letters.

The leaf biomass of the *Festuca* line EB15 was significantly affected by salinity, *Epichloë*, *Fusarium*, and the [*Epichloë* × *Fusarium*] interaction (Table 1). Leaf biomass decreased with salinity, E^+^ clones had greater biomass than E^–^ clones regardless of salinity, and inoculation with *Fusarium* increased the leaf biomass in all treatments, but this response was greater in E^–^ than in E^+^ plants (Figure 2A). In addition, root biomass was significantly greater in plants inoculated with *Fusarium* (0.818 ± 0.051 g vs. 0.647 ± 0.050 g) regardless of *Epichloë* infection or the salinity treatment (Table 1; Figure 2B).

**Table 1:** ANOVA results showing the effect of *Epichloë* presence, inoculation, and salt treatment on different parameters of *F. rubra* subsp. *pruinosa* plants.

		*Epichloë* (E)	Root endophyte (R)	Salt	R × Salt	E × R	E × Salt	E × R × Salt
**Shoot biomass**								
Line EB15/*F. oxysporum* T48	F_7,39_	6.08	29.5	4.64	1.65	8.06	0.632	0.061
	*p*	**0.019**	**<0.001**	**0.039**	0.208	**0.008**	0.432	0.808
Line TH12/*P. macrospinosa* T131	F_7,39_	0.03	14.3	3.78	2.42	0.092	0.429	1.78
	*p*	0.862	**<0.001**	0.061	0.129	0.763	0.517	0.191
**Root biomass**								
Line EB15/*F. oxysporum* T48	F_7,39_	1.65	5.51	0.004	0.464	0.079	2.89	0.005
	*p*	0.207	**0.025**	0.951	0.500	0.780	0.099	0.940
Line TH12/*P. macrospinosa* T131	F_7,39_	0.362	20.3	1.67	0.514	0.001	7.44	5.67
	*p*	0.552	**<0.001**	0.205	0.478	0.980	**0.010**	**0.023**
**Proline**								
Line EB15/*F. oxysporum* T48	F_7,23_	2.04	0.246	187	0.437	0.953	2.44	0.869
	*p*	0.175	0.628	**<0.001**	0.519	0.346	0.141	0.367
Line TH12/*P. macrospinosa* T131	F_7,23_	0.418	1.21	694	0.101	0.705	0.262	0.783
	*p*	0.529	0.291	**<0.001**	0.756	0.416	0.617	0.392
**Na**								
Line EB15/*F. oxysporum* T48	F_7,39_	1.69	40.1	193	41.1	10.6	2.01	9.16
	*p*	0.214	**<0.001**	**<0.001**	**<0.001**	**0.006**	0.178	**0.009**
Line TH12/*P. macrospinosa* T131	F_7,39_	0.128	9.25	622	8.45	6.9	0.379	6.75
	*p*	0.726	**0.009**	**<0.001**	**0.012**	**0.021**	0.549	**0.022**
**K**								
Line EB15/*F. oxysporum* T48	F_7,39_	0.429	0.005	25.5	0.096	0.005	0.663	0.663
	*p*	0.523	0.946	**<0.001**	0.761	0.944	0.429	0.429
Line TH12/*P. macrospinosa* T131	F_7,39_	0.357	0.163	24.4	0.682	0	1.31	0.001
	*p*	0.561	0.693	**<0.001**	0.424	0.997	0.273	0.988
**Na:K**								
Line EB15/*F. oxysporum* T48	F_7,39_	3.74	86.2	414	87.6	25.6	4.06	22.1
	*p*	0.074	**<0.001**	**<0.001**	**<0.001**	**<0.001**	0.064	**<0.001**
Line TH12/*P. macrospinosa* T131	F_7,39_	0.355	8.12	319	7.44	4.97	0.146	4.87
	*p*	0.561	**0.014**	**<0.001**	**0.017**	**0.044**	0.709	**0.046**
**Antioxidant capacity**								
Line EB15/*F. oxysporum* T48	F_7,39_	6.08	29.5	4.64	1.65	8.06	0.63	0.06
	*p*	**0.019**	**<0.001**	**0.039**	0.208	**0.008**	0.432	0.808
Line TH12/*P. macrospinosa* T131	F_7,39_	0.03	14.3	3.78	2.42	0.092	0.429	1.78
	*p*	0.862	**<0.001**	0.061	0.129	0.763	0.517	0.191
**Phenolic compounds**								
Line EB15/*F. oxysporum* T48	F_7,39_	1.65	5.51	0.004	0.464	0.079	2.89	0.005
	*p*	0.207	**0.025**	0.951	0.500	0.78	0.099	0.940
Line TH12/*P. macrospinosa* T131	F_7,39_	0.362	20.3	1.67	0.514	0.001	7.44	5.67
	*p*	0.552	**<0.001**	0.205	0.478	0.98	**0.010**	**0.023**

*Statistically significant values (p < 0.05) are shown in bold type*.

In *Festuca* line TH12, only *Periconia* had a significant effect on leaf biomass (Table 1). Inoculated plants (0.536 ± 0.049 g) had greater leaf biomass than uninoculated (0.331 ± 0.028 g), regardless of *Epichloë* and salinity (Figure 2C). Root biomass was significantly affected by *Periconia*, [*Epichloë* × salt] and [*Epichloë* × *Periconia* × salt] interactions (Table 1). The triple interaction indicated that the positive effect of *Periconia* on root growth was more pronounced in E^+^ plants at 0 mM NaCl and in E^–^ plants at 600 mM NaCl (Figure 2D).

### Sodium and Potassium Content

**Figure 3:**
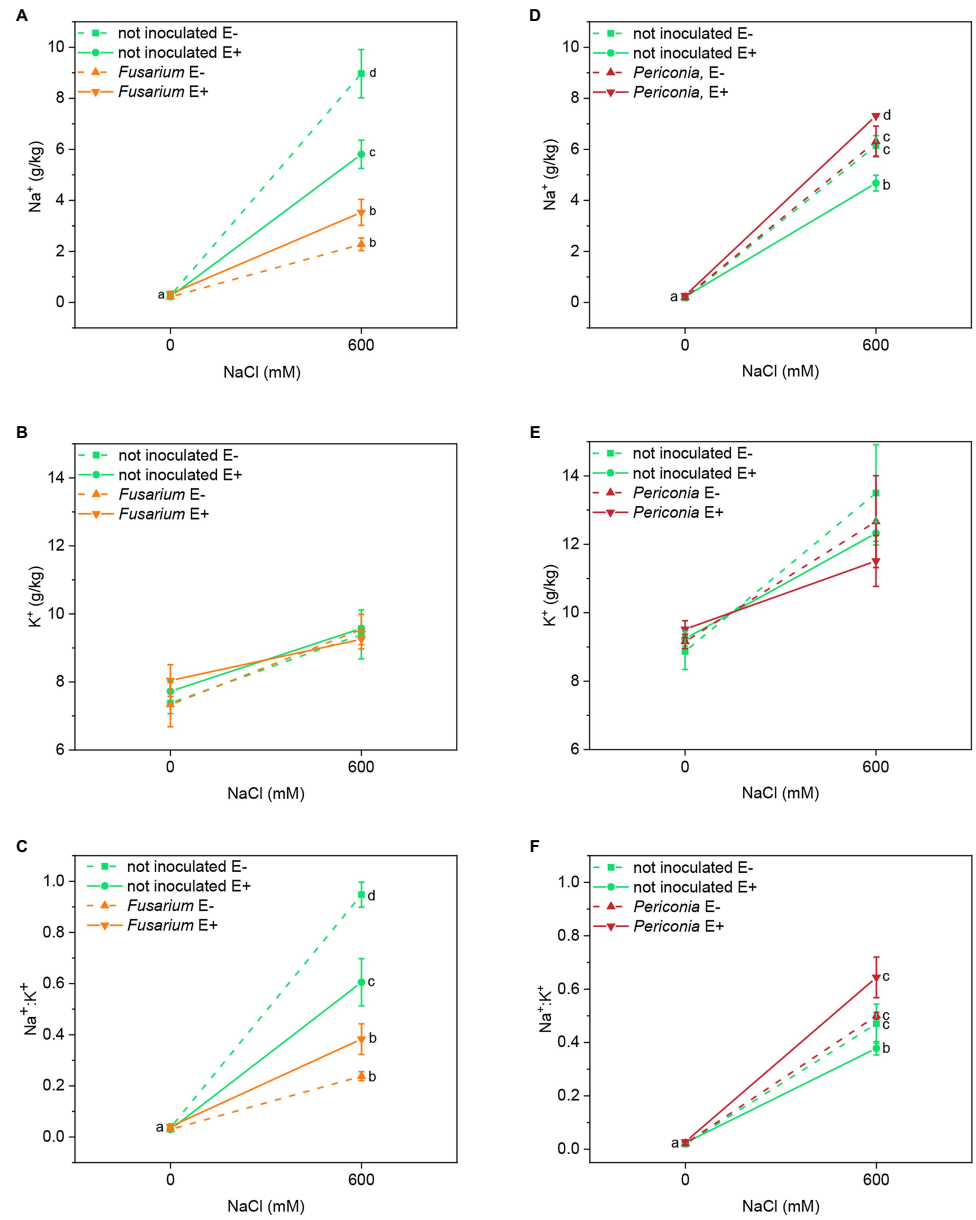
Leaf sodium and potassium content and Na^+^:K^+^ ratio in two FRP lines, EB15 (A–C) and TH12 (D–F). Each line was composed by clones symbiotic with E. festucae (E^+^) or Epichloë free (E^–^). Plants of each line were inoculated with F. oxysporum T48 (orange), P. macrospinosa T131 (dark red), or uninoculated (green), and subjected to two different salinity treatments (0 and 600 mM NaCl). Where a significant [salinity × Epichloë × root endophyte] interaction occurred, different means are indicated by different letters.

In both FRP lines, a significant effect of salinity, both root endophytes, and the interactions [*Epichloë* × root endophyte], [root endophyte × salinity], and [*Epichloë* × root endophyte × salinity] on Na^+^ content of leaves were detected (Table 1). In both plant lines, Na^+^ content increased in plants treated with 600 mM NaCl, and in uninoculated plants, it was lower in E^+^ than in E^–^ clones (Figures 3A,D). In *Festuca* line EB15, the Na^+^ content under salinity decreased significantly in plants inoculated with *Fusarium*, but it was affected by the *Epichloë* status. In line TH12, the opposite occurred, and at 600 mM NaCl, the Na^+^ content increased in E^+^ plants inoculated with *Periconia*; however, in E^–^ plants, differences between inoculation treatments were not significant (Figure 3D).

In both plant lines, the K^+^ content significantly increased at 600 mM NaCl, although this increase was less pronounced than the one observed for Na^+^ (Figures 3B,E). Neither the *Epichloë* status nor the root endophytes significantly affected this parameter (Table 1).

Mainly driven by the Na^+^ response, the Na^+^/K^+^ ratio significantly increased in leaves of all plants at 600 mM NaCl. In both lines, a significant effect of root endophyte inoculation [*Fusarium* and *Periconia*] and the interactions [*Epichloë* × root endophyte], [root endophyte × salinity], and [*Epichloë* × root endophyte × salinity] were detected (Table 1). In uninoculated plants of the line EB15 subject to salinity, this ratio was significantly lower in E^+^ than in E^–^ plants, and in plants inoculated with *Fusarium*, Na^+^/K^+^ further decreased in both E^+^ and E^–^ (Figure 3C). In line TH12, the Na^+^/K^+^ was also lower in uninoculated E^+^ plants at 600 mM NaCl, and the inoculation with *Periconia* did not reduce this ratio, as observed with *Fusarium* in the other line (Figure 3F).

### Proline Content

**Figure 4:**
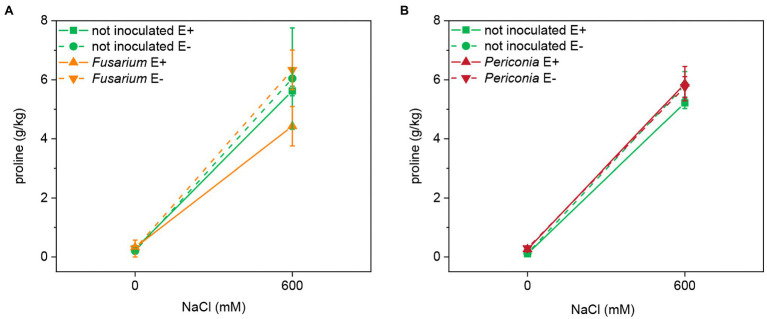
Leaf proline content at two different salinity treatments (0 and 600 mM NaCl) in two FRP lines, EB15 (A) and TH12 (B), each one composed by clones symbiotic with E. festucae (E^+^) or Epichloë free (E^–^), and inoculated with F. oxysporum T48 (orange) or P. macrospinosa T131 (dark red), or uninoculated (green).

Salinity had a significant effect on the leaf proline content in both FRP lines (Table 1; Figure 4). Neither *Epichloë* nor the root endophytes affected the content of this osmolyte. The proline content in leaves at 600 mM NaCl was very similar in both lines (EB15: 5.606 ± 0.440 g/kg; TH12: 5.664 ± 0.193 g/kg).

### Antioxidant Capacity and Phenolic Compounds Content

**Figure 5:**
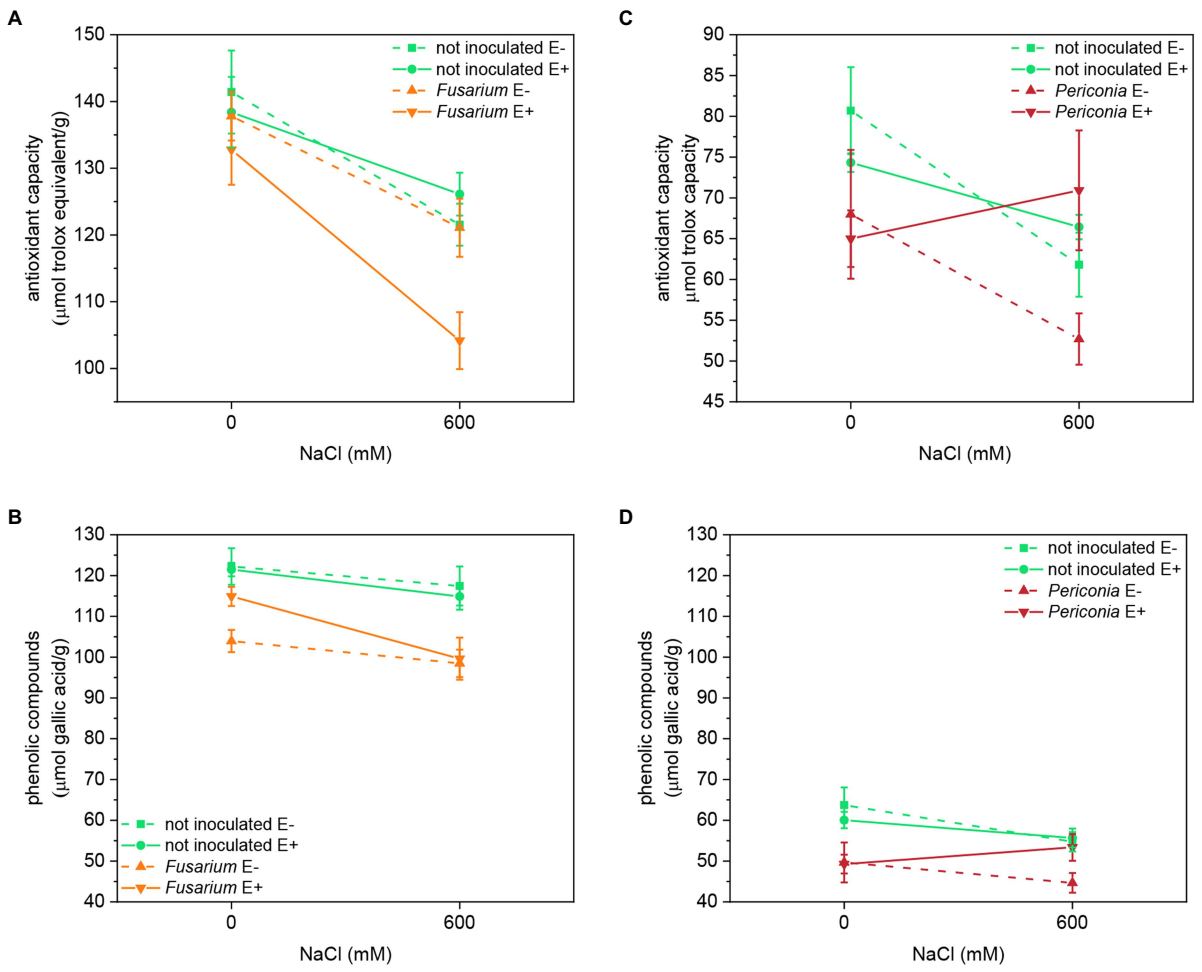
Antioxidant capacity and phenolic compounds content in two FRP lines, EB15 (A,B) and TH12 (C,D). Each line was composed by clones symbiotic with E. festucae (E^+^) or Epichloë free (E^–^). Plants of each line were inoculated with *F. oxysporum* T48 (orange), *P. macrospinosa* T131 (dark red), or uninoculated (green), and subjected to two different salinity treatments (0 and 600 mM NaCl).

Both salinity and *Fusarium* factors had a significant effect on the antioxidant capacity and content of total phenolic compounds (TPhC; Table 1; Figure 5). Plants inoculated with *Fusarium* had lower antioxidant capacity and TPhC content than uninoculated plants, and both parameters decreased with salinity. *Fusarium* had a significant interaction with *Epichloë*, and in inoculated plants, the antioxidant capacity was lower in E^+^ than in E^–^ plants.

The antioxidant capacity of FRP line TH12 was significantly affected by salinity and the [*Epichloë* × salinity] interaction (Table 1; Figure 5), decreased with salinity in E^–^ plants, but in E^+^ plants, differences between salt treatments were not statistically significant. Inoculation with *Periconia* was the only factor affecting the TPhC content, which significantly decreased in plants infected with the root endophyte, regardless of the *Epichloë* infection and the salt treatment (Table 1; Figure 5).

### Ergovaline Content

**Figure 6:**
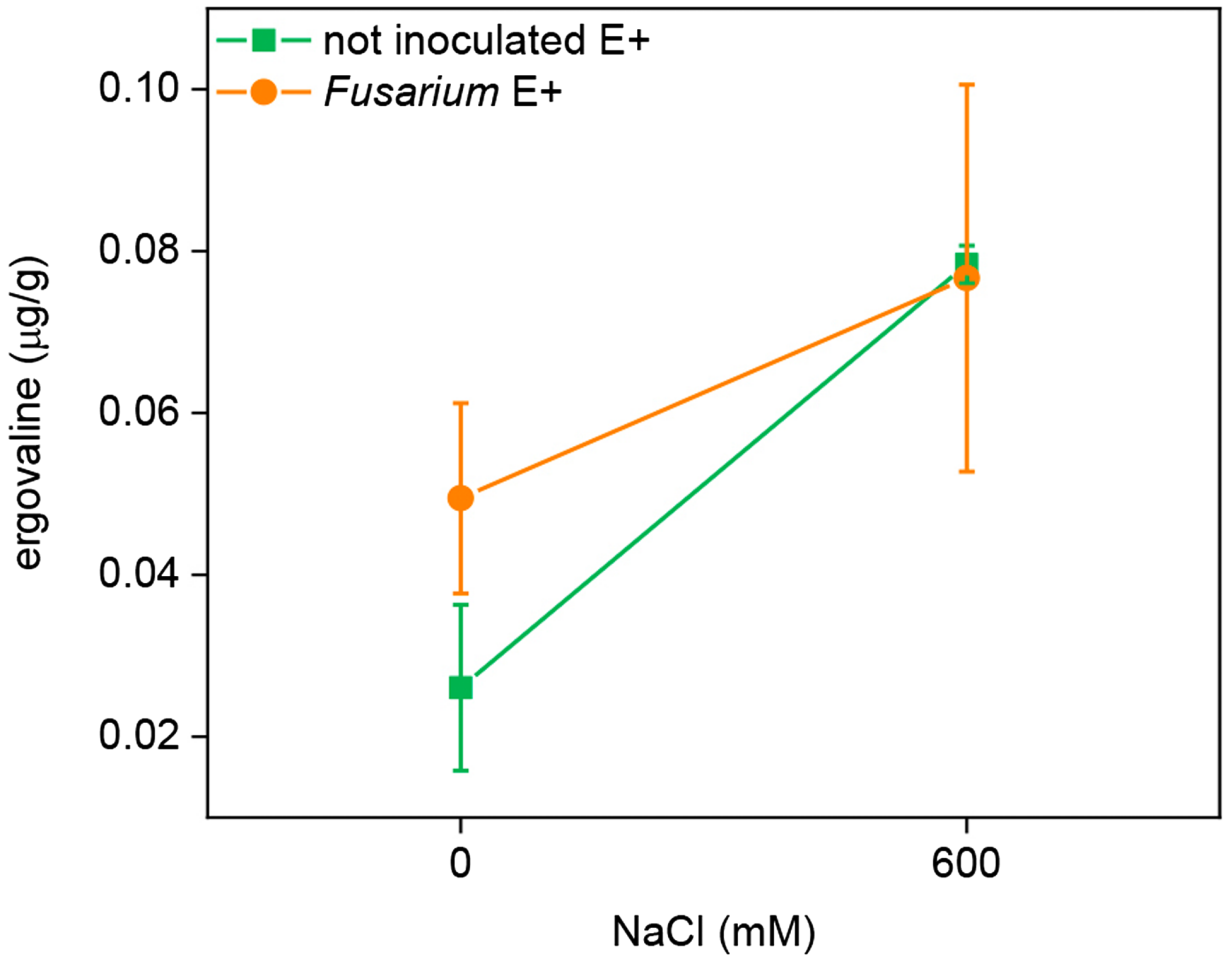
Ergovaline concentration in leaves of FRP line EB15 symbiotic with *E. festucae* (E^+^), inoculated with *F. oxysporum* T48 (orange) or uninoculated (green), and subjected to two different salinity treatments (0 and 600 mM NaCl).

*Epichloë festucae* can produce ergovaline in symbiotic FRP plants. Therefore, this alkaloid was analyzed only in E^+^ plants of each line. Plants inoculated with *Periconia* or uninoculated had ergovaline contents below the limit of quantification (0.02 μg/g), suggesting that this FRP-*Epichloë* combination does not produce ergovaline. In plants of line EB15, there was a significant positive effect of salinity (*F* = 6.02, *p* = 0.044) on ergovaline content, but the effect of *Fusarium* was not statistically significant (Figure 6).

### Root Microscopy

**Figure 7:**
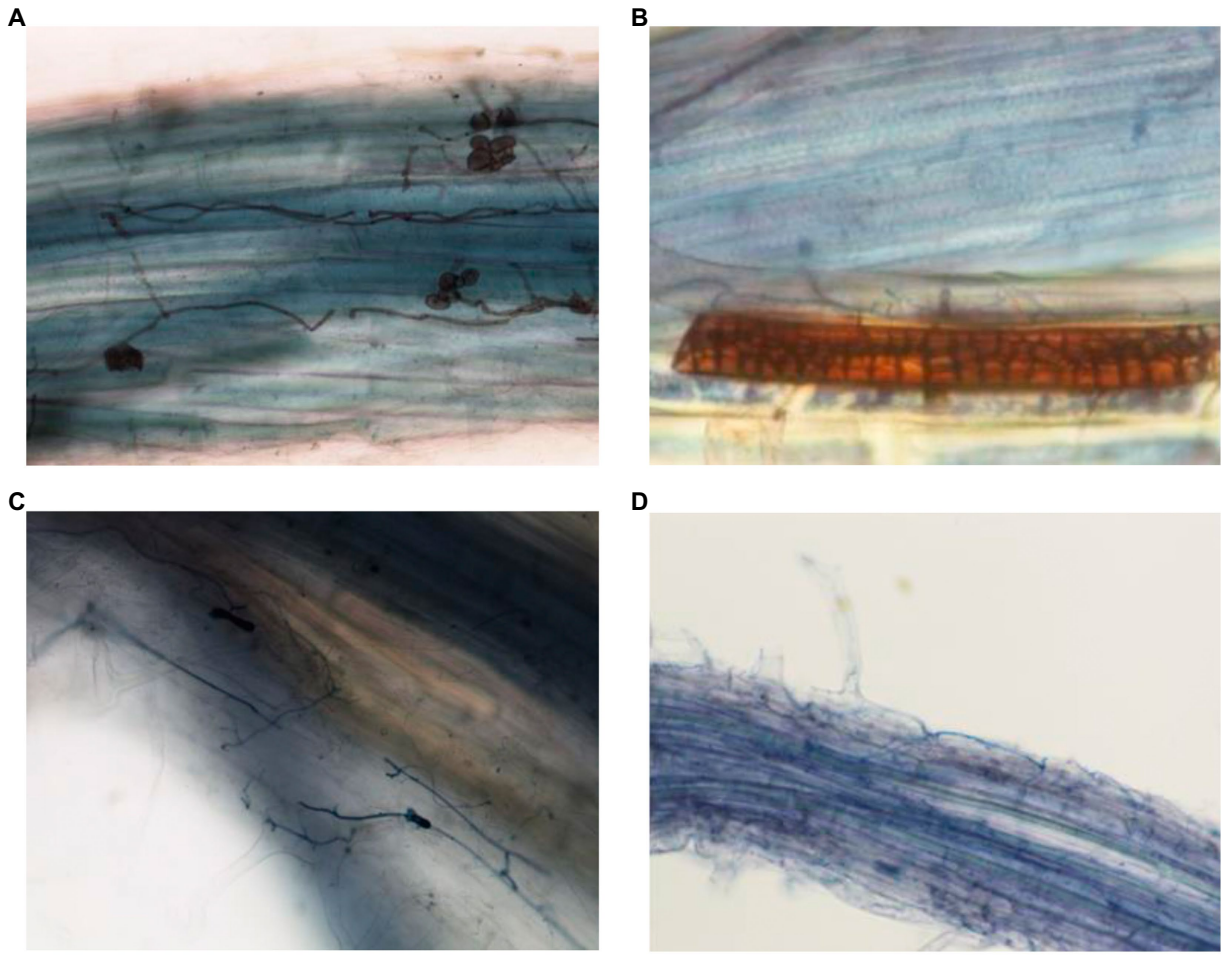
Fungal structures observed by light microscopy in roots of plants inoculated with *P. macrospinosa* **(A,B)** and *F. oxysporum* **(C,D)**. In roots inoculated with *Periconia*, melanized septate hyphae and spores **(A)** and microsclerotia **(B)** are visible. In *Fusarium*-inoculated plants, hyphae are visible in the root cortex.

The presence of fungal structures in inter and intracellular spaces indicated the presence of the root endophytes and successful plant inoculation (Figure 7). In roots of plants inoculated with *Periconia*, melanized septate hyphae and microsclerotia were observed (Figures 7A,B). These structures are typical of DSEs. In plants inoculated with *Fusarium* T48, hyphae were observed in the root cortex (Figures 7C,D).

### *In vitro* Interaction Between *Epichloë festucae* and Root Endophytes

**Figure 8:**
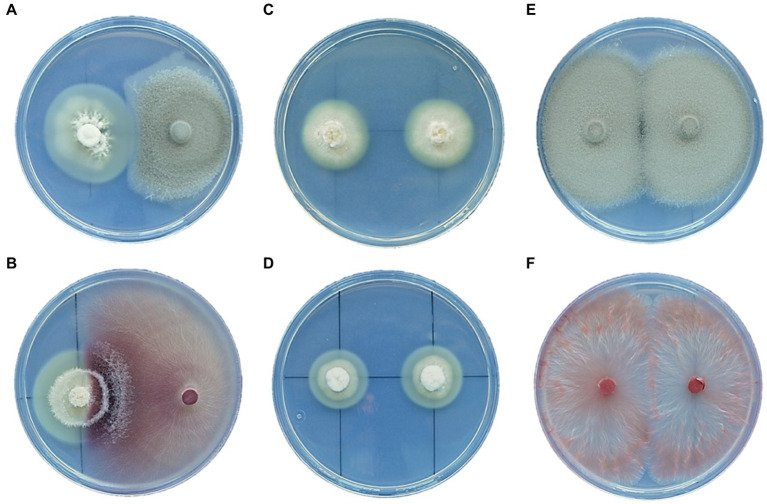
Dual-culture interaction between *E. festucae* and the root endophytes *P. macrospinosa* **(A)** and *F. oxysporum* T48 **(B)**. Dual-culture control of *Epichloë* EB15 **(C)**, *Epichloë* TH12 **(D)**, *P. macrospinosa* T131 **(E)**, and *F. oxysporum* T48 **(F)**. All cultures are two weeks old.

Dual cultures of *E. festucae* strains isolated from the FRP lines and the root endophytes *P. macrospinosa* and *F. oxysporum* were established. The results showed that *E. festucae* had an inhibitory effect on the mycelial growth of *P. macrospinosa* (Figure 8A). However, this pattern was not observed with *F. oxysporum*, whose mycelium grew over the *E. festucae* colony (Figure 8B).

In dual cultures with *Periconia* and *Fusarium*, the radial growth of *E. festucae* was stimulated, increasing by 42 and 48%, respectively, relative to the dual cultures of *E. festucae* alone (Figures 8C,D).

## Discussion

### Plant Characteristics Related to Salinity Tolerance

The halophytic character of FRP was clearly observed in its response to salinity. The biomass reduction caused by exposure to high salinity was low, being statistically significant only for the leaf biomass of line EB15, while the root growth was not significantly affected in any case. Regarding this, shoot growth is reported as more sensitive to salinity than root growth in *F. rubra*, as well as in other plant species (Baumeister and Merten, 1981; Munns and Tester, 2008).

The response of ion and solute accumulation to salinity stress gives an insight into some mechanisms of adaptation of FRP to its natural environment in sea cliffs. As previously reported for *F. rubra* subsp. *litoralis* (Rozema et al., 1978), exposure to salinity caused a substantial increment in the shoot content of Na^+^ and proline in FRP. This type of response occurs in plant species having tissue tolerance to Na^+^. In contrast to the alternative mechanism of Na^+^ exclusion from leaf blades, plants having tissue tolerance accumulate Na^+^ in their tissues by sequestering it into cell vacuoles. When this occurs, an osmotic adjustment of the cytoplasm is needed to maintain cell turgor, and this is achieved by the synthesis and accumulation of proline or other compatible solutes, as well as K^+^ (Flowers and Colmer, 2008; Munns and Tester, 2008; Zhao et al., 2020). This balance with proline, a common osmolyte among halophytic grasses (Slama et al., 2015), occurred in FRP regardless of the presence of leaf or root symbionts. A significant increase in K^+^ leaf concentration also occurred in response to salinity, independently of the symbiotic status of the plants. Different responses in terms of K^+^ content have been observed in different halophytes; some maintain the K^+^ concentration regardless of salinity, while others, including some maritime halophytes like FRP, show increased K^+^ content under salinity as a stress tolerance strategy (Flowers and Colmer, 2008; Assaha et al., 2017).

Both FRP lines differed in salinity tolerance. Salinity had a significant negative effect on the leaf biomass of line EB15, but not on TH12. At the same time, the Na^+^ content of line TH12 was lower than that of EB15. A similar inverse relation between salinity tolerance and Na^+^ content was reported to occur among subspecies of *F. rubra*, with the most tolerant subspecies having less Na^+^ content than the least tolerant inland subspecies (Rozema et al., 1978). Tolerance to salinity is an inheritable character in *F. rubra* (Humphreys, 1982), and the mechanisms related to the management of cellular Na^+^ are very likely to be related to it.

### *Epichloë* Effects on Plants Subject to Salinity

Because of salinity, scarcity of soil and nutrients, and frequent wind exposure, the FRP habitat in sea cliffs could be considered suboptimal for plant growth. Considering that the incidence of *E. festucae* is relatively high in FRP populations, about 66% (Pereira et al., 2019), it could be expected that in such demanding habitat the benefits from this symbiosis must be greater than its costs for the plant in order to support such incidence rates in a vertically inherited symbiont.

While some works report a clearly beneficial effect of *Epichloë* endophytes on several parameters related to salt tolerance in grasses (Song et al., 2015; Wang et al., 2020), others indicate that beneficial effects might be dependent on the grass-endophyte genotypes (Sabzalian and Mirlohi, 2010). In terms of leaf or root biomass, our results do not support an obvious or unambiguous role of *Epichloë* itself in FRP salt tolerance, confirming the results of a previous experiment (Zabalgogeazcoa et al., 2006b). However, the accumulation of Na^+^ was significantly lower in leaves of E^+^ than in E^–^ plants of both plant lines. Such reduction in Na^+^ accumulation under salinity might be advantageous for salinity tolerance and habitat adaptation. In fact, among subspecies of *F. rubra*, the tolerance to salinity appears to be inversely correlated to Na^+^ accumulation (Rozema et al., 1978). A similar effect of Na^+^ reduction in plants symbiotic with *Epichloë* was also reported in other grass species (Sabzalian and Mirlohi, 2010; Song et al., 2015). Nevertheless, other important fitness parameters, such as seed production or germination, have not been examined in relation to the *Epichloë* status of plants, and such information could help to understand the high prevalence of this symbiosis in sea cliff populations.

Plant protection against herbivores mediated by fungal alkaloids like ergovaline is often cited as a main driver of *Epichloë*-grass symbioses (Schardl et al., 2013). However, macroherbivores are absent from sea cliffs, and herbivore protection mediated by fungal alkaloids is not a satisfactory hypothesis to understand why *Epichloë* infection rates are high in sea cliff populations. It is worth noting that the incidence of *Epichloë* in *F. rubra* populations from sea cliffs is very similar to that observed in inland populations from semiarid grasslands (Zabalgogeazcoa et al., 1999; Pereira et al., 2019). Perhaps, other unknown factors than herbivory are as important for the favorable selection of these symbiotic associations.

Only plants of the line EB15 contained the fungal alkaloid ergovaline. Phenotypic variation in ergovaline content occurs among FRP plants, and in a previous survey, 21% of the plants analyzed did not contain this alkaloid (Vázquez de Aldana et al., 2007). Lack of ergovaline in symbiotic plants could be due to the absence of functional genes in the biosynthetic pathway, plant-fungal compatibility, environmental effects, or both (Schardl et al., 2013; Vázquez de Aldana et al., 2020b). The ergovaline content increased greatly in EB15 plants subject to salinity. Increased content of diverse alkaloids in response to salinity has been reported in several plant species (Wang et al., 2010; Li et al., 2020), including some ergot alkaloids produced by *Epichloë* in the grass *Achnatherum inebrians* (Zhang et al., 2011). Whether *Epichloë* alkaloids could have a function other than defensive in plants is unknown.

The antioxidant capacity of FRP plants under salinity stress did not differ in response to *Epichloë* symbiosis itself (in the absence of root endophytes). This result contrasts with those of Chen et al. (2018), who observed an increment in total antioxidant activity in E^+^ plants of *Hordeum brevisubulatum* subject to salinity. The fact that the functional intensity of the antioxidant machinery is time dependent, and several enzymes peak soon after stress exposure, but later return to a basal level (Baltruschat et al., 2008), might explain this difference. Alternatively, FRP is a halophytic marine plant species and has an efficient endogenous machinery to cope with salinity, where accumulation of antioxidants might not be responsible for the salt stress adaptation.

### Root Endophyte Effect on Plants Subject to Salinity

The most remarkable results from this study are the observation that both *F. oxysporum* and *P. macrospinosa*, prevalent root endophytes in natural populations of FRP, have a significant beneficial effect on plant growth, regardless of the salinity treatment. Thus, these two fungal components of the FRP microbiome could favor plant hosts in their native habitat. *Fusarium oxysporum* is the most abundant fungal species detected in the root endosphere of FRP, with a prevalence of 57% in natural populations, and is a likely component of the core microbiome of this grass (Pereira et al., 2019). Asymptomatic root infections like the ones we observed in FRP plants are common for many *Fusarium* species (Bacon and Yates, 2006). *Fusarium oxysporum* is best known for its pathogenic *formae speciales* but also contains numerous strains having an endophytic lifestyle, like those present in FRP roots (Demers et al., 2015; Edel-Hermann and Lecomte, 2019). Endophytic, non-pathogenic, *F. oxysporum* strains may act as biocontrol agents against several root pathogens, including pathogenic strains of their own species (Constatin et al., 2020; de Lamo and Takken, 2020), and in some circumstances might promote plant growth in the absence of disease (Bitas et al., 2015).

Root endophytes, like *Serendipita indica* or *Fusarium culmorum*, have been reported to increase host plant tolerance to salinity (Waller et al., 2005; Rodriguez et al., 2008). The latter is a dominant endophyte in several organs of the beach grass *Leymus mollis*, and probably, it is an important microbiome component for the adaptation of the plants to their maritime habitat (Rodriguez et al., 2008). In view of our results, a similar function could be expected from *F. oxysporum* endophytes in FRP plants.

A parameter having a remarkable response in FRP plants inoculated with *F. oxysporum* was the Na^+^ content of leaves, which showed a pronounced decrease when compared to uninoculated plants. This response did not occur in plants inoculated with *P. macrospinosa*. As explained before, the results from our experiment suggest that in the absence of *F. oxysporum*, FRP plants cope with salinity by means of a tissue tolerance mechanism consisting of the vacuolar sequestration of Na^+^. However, plants also have other mechanisms to cope with salinity, like the exclusion of Na^+^ from entering the plants, a mechanism centered in plant roots (Munns and Tester, 2008; Zhao et al., 2020). Thus, the association with *F. oxysporum* could be limiting Na^+^ uptake from plant roots by means of modulating or complementing the plant Na^+^ exclusion machinery. A similar situation of symbiont-mediated Na^+^ exclusion has been reported in *Arabidopsis* inoculated with *S. indica* (Abdelaziz et al., 2019; Lanza et al., 2019).

In addition, the growth promotion observed in the absence of salt in plants inoculated with *F. oxysporum* might be related to nutrient acquisition, an important issue in a habitat where soil is scarce or absent. Improved nutrient acquisition mediated by symbiotic fungi could occur in several ways; for instance, root endophytes could help to recycle dead plant material (Upson et al., 2009; Vázquez de Aldana et al., 2013), produce plant hormones that stimulate root growth (Sirrenberg et al., 2007), or alter the chemistry or microbiota of the rhizosphere (Alegría Terrazas et al., 2016). Thus, the increased shoot biomass observed in inoculated plants could be due to the greater root size caused by hormonal stimulation, increased nutrient availability, or both together mediated by *F. oxysporum*.

*Periconia macrospinosa* is a DSE with a wide host range, which has been described in roots of numerous grasses and other plants (Mandyam et al., 2012). In natural populations of FRP, the incidence of this taxon was about 16%, being one of its most abundant root endophytes (Pereira et al., 2019). The search for the ecological function of DSE symbioses has been elusive, although some studies report increased biomass and nutrient (N, P) content, as well as salt tolerance in host plants (Mandyam and Jumpponen, 2005; Newsham, 2011; Gonzalez Mateu et al., 2020). In the present study, *P. macrospinosa* caused a significant increase in leaf and root biomass of its original host plant, FRP, in the presence as well as in the absence of salinity stress. *Periconia macrospinosa* is known to produce a wide range of extracellular enzymes able to metabolize numerous substrates, organic as well as inorganic (Mandyam et al., 2010; Knapp and Kovács, 2016). This fungus, which is thought to have a life cycle as a latent saprobe, could be involved in nutrient cycling and mobilization (Yakti et al., 2018). In the habitat of FRP plants, where soil is often nonexistent, nutrient cycling from dead plants or other organic remains could be very important for habitat adaptation. In contrast with *F. oxysporum*, this fungus did not reduce the Na^+^ content of the plants; therefore, as a symbiont, its contribution might not be related to salinity tolerance.

### Interactions Among Holobiont Components

*Festuca rubra* is a mainly outcrossing species that can also reproduce asexually by means of vegetative tillers produced from rhizomes (Harberd, 1961). Thus, populations of *F. rubra* could be conceived as groups of genotypically distinct individuals, some of which could be more or less represented by means of clonal expansion. As we found in this study, distinct plant genotypes can differ in their tolerance to salinity, an important trait for adaptation to sea cliffs. *Epichloë festucae* endophytes interact with plant genotypes in *Festuca* populations. As observed, the response to salinity of the plant individuals can be modified by their interaction with *Epichloë* endophytes, which reduce their Na^+^ accumulation. Thus, the adaptation to salinity of both plant genotypes might be augmented by symbiosis with *Epichloë*. Further interactions seemed to occur with root endophytes. For instance, in terms of root growth and Na^+^ accumulation, *P. macrospinosa* was positive for E^–^, but not for E^+^ plants, and the trend seemed to be opposite in the case of *F. oxysporum*. In addition, although *Epichloë* and root endophytes occupy different plant compartments, the dual-culture experiments suggested that *E. festucae* cultures respond to the presence of both root endophytes. In these experiments, the growth of *Epichloë* was stimulated by the presence of the root endophytes, and *Periconia* was inhibited by *Epichloë*, but *Fusarium* was not. Taking into account that more than 100 species of culturable fungi have been identified in FRP roots (Pereira et al., 2019), multiple interactions among microbiome components are likely to have important effects on distinct plant genotypes. Such a complex landscape of interactions affecting holobiont performance might help to understand why FRP populations having a 100% incidence of *E. festucae* are rare in marine and inland ecosystems. Even if *E. festucae* is beneficial for some symbiotic plants, other holobiont configurations might compensate for its absence.

## Conclusion

This study sheds light on how a plant supported by its microbiome can adapt to an inhospitable habitat in sea cliffs. To cope with salinity, FRP seems to rely on a tissue tolerance mechanism that allows its cells to accumulate Na^+^, possibly in vacuoles, and an osmotic counterbalance occurs in the cytoplasm by means of proline and K^+^. In addition to these intrinsic plant mechanisms, *E. festucae*, *F. oxysporum*, and *P. macrospinosa*, three fungal endophytes highly prevalent in natural populations of *F. rubra* and also contributed to improve plant performance under salinity. *Epichloë* caused a Na^+^ reduction in leaves under salinity, which might be associated with salinity tolerance and plant survival in the long term. *Fusarium oxysporum*, the most abundant root endophyte from *F. rubra*, appears to contribute two different adaptive functions to symbiotic plants: first, promotion of the growth of leaves and roots in the presence as well as in the absence of salinity, and second, it caused a decrease in leaf Na^+^ content under salinity, a function which as above suggested for *Epichloë* could improve plant adaptation to salinity. *Periconia macrospinosa* promoted the growth of leaves and roots of *F. rubra* plants regardless of the salinity treatment. Although the mechanisms mediating growth promotion or salinity tolerance are unknown, each of these three components of the *F. rubra* core mycobiome contributed different functions, which are beneficial for plant adaptation to its habitat in sea cliffs, supporting our initial hypothesis.

## Data Availability Statement

The raw data supporting the conclusions of this article will be made available by the authors, without undue reservation.

## Author Contributions

EP designed and made the experiments, performed the chemical analyses, root microscopy, and dual-culture assays, and analyzed the data. All authors worked in the design of experiments, analysis of data, and writing of the manuscript.

### Conflict of Interest

The authors declare that the research was conducted in the absence of any commercial or financial relationships that could be construed as a potential conflict of interest.
